# Hidradénome papillifère

**DOI:** 10.11604/pamj.2014.17.274.4177

**Published:** 2014-04-14

**Authors:** Rajaa Elouarradi, Ouafa Hocar

**Affiliations:** 1 Service de Dermatologie, CHU Mohamed VI, faculté de Médecine et Pharmacie de Marrakech, Université Cadi Ayyad, Maroc

**Keywords:** Hidradénome papillifère, nodule, tumeur, hidradenoma papilliferum, nodule, tumor

## Image en medicine

C'est une tumeur annexielle bénigne à différentiation sudorale rare, qui touche la femme adulte. Elle peut siéger au niveau des grandes lèvres ou la région périnéale. Sur le plan clinique c'est un petit nodule (quelques mm), souvent unique, mobile, élastique, de couleur rose ou bleutée, parfois végétant et hémorragique ou pédiculée. Sur le plan histologique c'est une tumeur kystique encapsulée, située dans le derme profond, sans connexion avec l’épiderme. Cette tumeur est remplie de villosités conjonctives, et la lumière est tapissée de deux assises cellulaires, une assise sécrétrice et une assise de petites cellules cuboïdes à noyaux très basophiles (cellules myoépithéliales). Le diagnostic différentiel est posé devant un kyste wolfien ou une glande mammaire aberrante. Le traitement repose sur l'exérèse chirurgicale.

**Figure 1:**
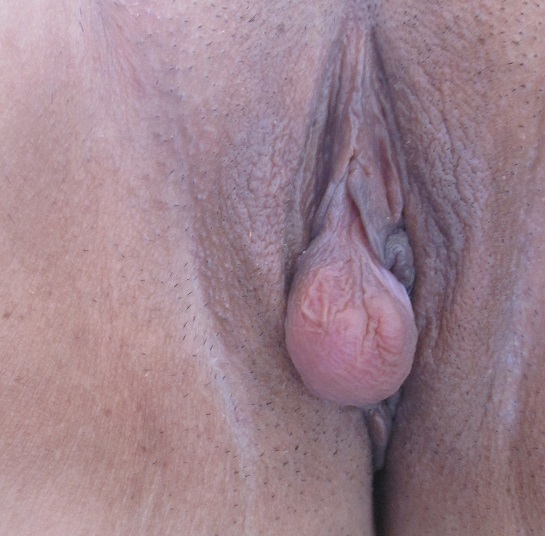
Hidradénome papillifère de localisation vulvaire

